# Fog Computing: Enabling the Management and Orchestration of Smart City Applications in 5G Networks

**DOI:** 10.3390/e20010004

**Published:** 2017-12-23

**Authors:** José Santos, Tim Wauters, Bruno Volckaert, Filip De Turck

**Affiliations:** Ghent University - imec, IDLab, Department of Information Technology, Technologiepark-Zwijnaarde 15, B-9052 Ghent, Belgium

**Keywords:** fog computing, 5G, IoT, smart cities, management and orchestration

## Abstract

Fog computing extends the cloud computing paradigm by placing resources close to the edges of the network to deal with the upcoming growth of connected devices. Smart city applications, such as health monitoring and predictive maintenance, will introduce a new set of stringent requirements, such as low latency, since resources can be requested on-demand simultaneously by multiple devices at different locations. It is then necessary to adapt existing network technologies to future needs and design new architectural concepts to help meet these strict requirements. This article proposes a fog computing framework enabling autonomous management and orchestration functionalities in 5G-enabled smart cities. Our approach follows the guidelines of the European Telecommunications Standards Institute (ETSI) NFV MANO architecture extending it with additional software components. The contribution of our work is its fully-integrated fog node management system alongside the foreseen application layer Peer-to-Peer (P2P) fog protocol based on the Open Shortest Path First (OSPF) routing protocol for the exchange of application service provisioning information between fog nodes. Evaluations of an anomaly detection use case based on an air monitoring application are presented. Our results show that the proposed framework achieves a substantial reduction in network bandwidth usage and in latency when compared to centralized cloud solutions.

## 1. Introduction

In recent years, the Internet of Things (IoT) has transformed objects of everyday life into communicating devices. The number of connected devices will be between 10 and 12 billion by 2021 [1], making it impossible for current network technologies to support this enormous growth. Future networked systems must adapt existing network architectures to future needs and design and develop new management capabilities to help meet the stringent requirements of future use cases. In fact, the upcoming 5G networks aim to tackle these new business opportunities by introducing very high carrier frequencies, an enormous number of antennas and new functionalities, such as Device-to-Device communication (D2D) and fog computing [2]. The fog computing paradigm, which places resources on the edges of the network, extends the cloud computing paradigm to deal with the eminent growth of connected devices [3]. Fog computing brings computing power, storage and memory capacity closer to wireless gateways, sensors and actuators since these devices are currently lacking in terms of such capacities [4]. The so-called Fog Nodes (FNs) are cloud entities with a small amount of computational resources distributed across the network that must be able to communicate with a different variety of devices and offer them solutions to gather, process and filter data [3,5]. These procedures imply a proper resource allocation of multiple services, which is not an easy task. Nowadays, resource provisioning and orchestration is present in different topics of literature related to the life-cycle management of applications and services. Although orchestration is relatively mature in data centers, in fog architectures, there is still a large number of research challenges associated with this approach since fog computing is still in the early stages and needs more time to evolve [6]. In fog architectures, orchestration should be distributed across the multiple FNs, which are then responsible for the local resource provisioning and deployment of applications and services, thereby ensuring the necessary Quality of Service (QoS).

In the last few years, IoT applications have been implemented as a set of small and independent micro-services. The micro-services architecture is a relatively new term in software patterns [7]. The micro-service paradigm is an extension of the traditional Service-Oriented Architecture (SOA) paradigm, where an application is decomposed into a set of fine-grained services. Each service communicates through lightweight communication protocols. Research studies have been carried out to solve the issues of abstracting end device functionalities, trying to provide a suitable architecture with service management and composition capabilities able to link a set of micro-services in a set of IoT applications. Each micro-service can be provided by a lightweight container, which may be used by multiple tenants. In a smart city scenario, resources should be distributed within the network ensuring that the micro-services that make up an application are allocated and instantiated close to the end device that is requesting the IoT application [8]. Multiple factors should be taken into account to ensure proper resource allocation such as latency, bandwidth, energy efficiency and cost.

In this article, a fog-based management and orchestration framework is proposed to deal with the application service placement problem in smart cities. Our approach follows the guidelines of the European Telecommunications Standards Institute (ETSI) Network Function Virtualization (NFV) Management and Orchestration (MANO) architecture, extending it with additional software components, which will offer not only high computing performance, but also monitoring and data analysis functionalities. Furthermore, based on our expertise in the network management domain, a novel fog protocol is foreseen to enable the exchange of application service information between FNs to provide fast service provisioning decisions for smart city applications. This way, each FN can decide where and when it is more suitable to deploy and instantiate each instance of the micro-services composing a smart city application. Therefore, an application layer Peer-to-Peer (P2P) fog protocol based on the Open Shortest Path First (OSPF) routing protocol is proposed to deal with the exchange of application service information between FNs and the cloud layer. Finally, the evaluation of an anomaly detection smart city use case based on an air monitoring application is presented. Our results show that the proposed fog computing framework achieves a substantial reduction in network bandwidth usage and in latency when compared to centralized cloud solutions.

The remainder of the article is organized as follows. In the next section, related work is discussed. Section 3 introduces the proposed fog computing management and orchestration framework. Then, in Section 4, the evaluation scenario is described, which is followed by the evaluation of the results in Section 5. Finally, conclusions are presented in Section 6.

## 2. Related Work

With the advent of 5G networks and by exploiting the advantages of new paradigms, such as NFV, Software-Defined Networking (SDN) and Machine-to-Machine (M2M) communication, autonomous network management functionalities become feasible. This section provides a summary of the standardization activities, research projects and open source initiatives relevant for the specification and implementation of a MANO framework for 5G-enabled smart cities.

### 2.1. Standardization Activities

This subsection provides a brief summary of the standardization activities relevant in the management and orchestration domain.

#### 2.1.1. ETSI oneM2M

**Figure 1:**
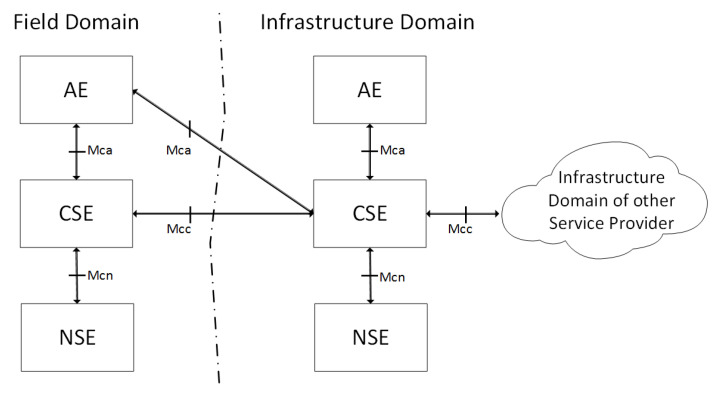
oneM2M functional architecture.

In January 2009, the ETSI M2M technical committee was established with the aim to develop an end-to-end high level architecture for M2M. ETSI oneM2M aims to establish for M2M what 3GPP realized for mobile networks. The final release has been available since 2016 [9], and the correspondent architecture is shown in Figure 1. The architecture consists of two distinct domains: the field domain and the infrastructure domain. The Application Entity (AE) is responsible for implementing an M2M application service logic in the application layer. Each execution instance is identified with a unique AE Identifier (AE-ID). Examples of the AEs include an instance of a remote blood sugar monitoring application, a power metering application or an emergency response application. The Common Services Entity (CSE) represents an instantiation of a set of common service functions of the M2M environments. These service functions are exposed to other entities through the AE – CSE (Mca) and CSE - CSE (Mcc) reference points. Each CSE is identified with a unique CSE-ID. Examples of service functions offered by CSEs include: data management, device management and M2M service subscription management. Finally, the Network Service Entity (NSE) provides services from the underlying network to the CSEs. Examples of such services include location services and device triggering [9]. The reference point CSE – NSE (Mcn) is used for accessing underlying NSEs. The proposed fog computing framework follows the guidelines of ETSI OneM2M regarding device management and security standards by including the correspondent components in our framework. Nevertheless, the definition of MANO functionalities and components in the ETSI OneM2M reference architecture are still in an early stage.

#### 2.1.2. OpenFog Consortium

**Figure 2:**
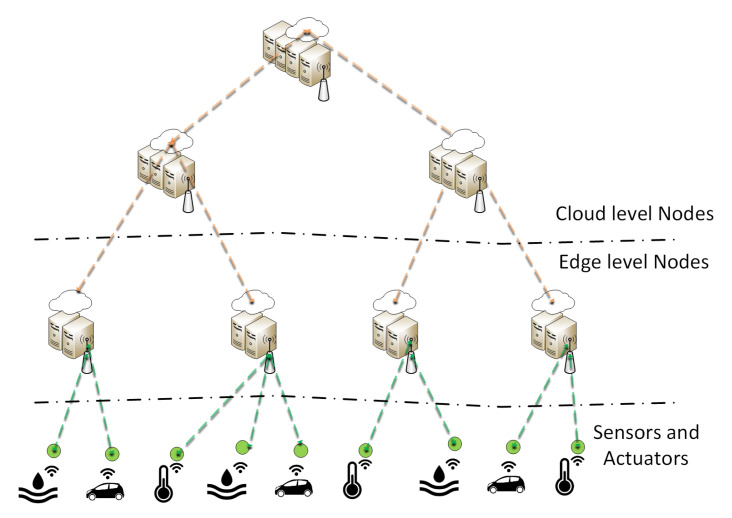
The OpenFog reference architecture: N-tier fog deployment.

The OpenFog Consortium is working on a framework for efficient and reliable networks between clouds, endpoints and services based on open standard technologies. Recently, OpenFog released a reference architecture [10] that clarifies the characteristics and the requirements for fog computing, focusing primarily on the FN. The OpenFog reference architecture for a multi-tier deployment is shown in Figure 2. However, the definition of MANO components in the OpenFog reference architecture are still superficial, and therefore, it is expected that the consortium will work in this direction in future specifications.

#### 2.1.3. ETSI MEC

**Figure 3:**
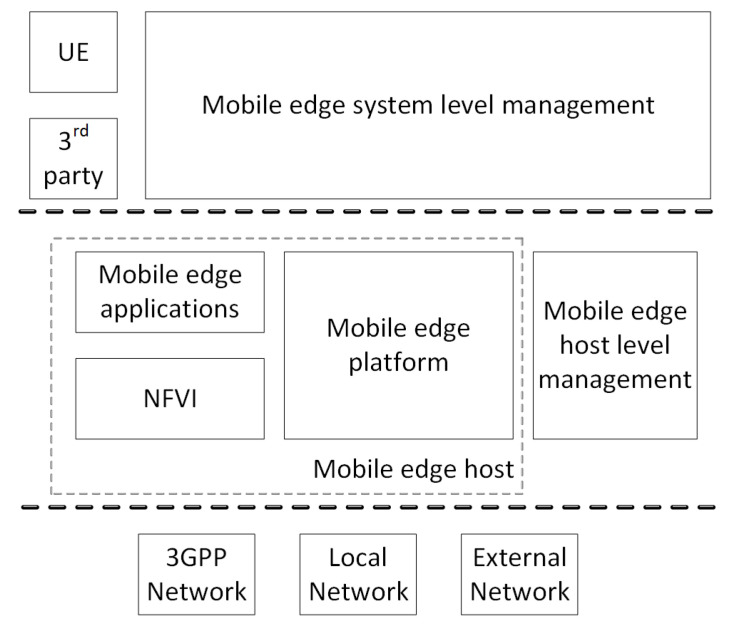
The mobile edge system reference architecture.

The ETSI Mobile Edge Computing (MEC) technical committee is currently working on a reference architecture [11] for an orchestrator with similar requirements to the orchestrator required by fog computing architectures. MEC is emphasizing the need to consider a set of stringent constraints, such as application instantiations and service reallocations, both very important requirements for fog solutions. The MEC framework is shown in Figure 3. MEC is focused on evolving the mobile network edges, in order to create a cloud environment close to the Radio Access Network (RAN) that hosts enhanced services provided by the Mobile Network Operator (MNO) or third parties. Mobile Edge (ME) applications run on top of a generic cloud infrastructure located within the RAN, referred to as the mobile edge host. The mobile edge host is an entity containing a mobile edge platform and a virtualization infrastructure, which provides compute, storage and network resources for the purpose of deploying mobile edge applications. The mobile edge platform is the collection of functionalities required to run mobile edge applications on a virtualization infrastructure enabling the provisioning and consumption of mobile edge services [11]. Nevertheless, a detailed and complete specification of the MEC Orchestrator reference architecture is still missing.

#### 2.1.4. ETSI NFV MANO

**Figure 4:**
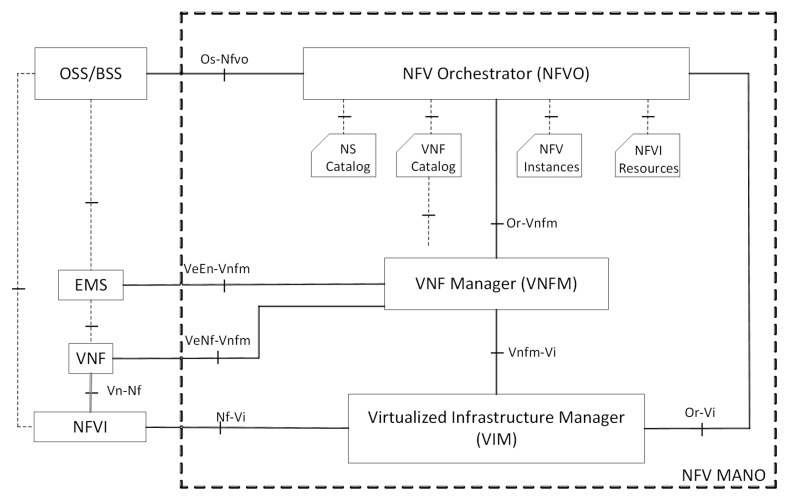
The NFV MANO architectural framework with reference points.

The ETSI NFV MANO technical committee is working on the standardization of management and orchestration frameworks required for the provisioning of Virtual Network Functions (VNFs) and Network Services (NSs). The MANO committee focuses on the definition of a reference architecture to overcome the challenges of the virtualization paradigm by covering operational and management aspects, such as service life-cycle workflows, information elements and interfaces. The final release has been available since 2014 [12], and the correspondent architecture is shown in Figure 4. This reference architecture allows network operators to apply a smooth transition to the new management paradigm, where legacy services and functionalities are gradually augmented with SDN and NFV capabilities. The ETSI NFV MANO architecture is composed of four main functional blocks:

- Operations Support System/Business Support System (OSS/BSS):
  - Responsible for the control of software applications used to support back-office activities;
  - Coordination of business operations, such as billing and customer services.
- NFV Orchestrator (NFVO):
  - Responsible for the registration of new VNFs and NSs;
  - Life-cycle management (including instantiation, scale-out/in, termination).
- VNF Manager (VNFM):
  - Life-cycle management of VNF instances;
  - Coordination of the main configurations between the NFV Infrastructure (NFVI) and the Elemental Management System (EMS).
- Virtualized Infrastructure Manager (VIM):
  - Controlling and managing the NFVI compute, storage and network resources;
  - Collection and forwarding of performance measurements and events;

The proposed fog computing framework in this article follows the ETSI NFV MANO reference architecture, extending it with additional software components. Our architectural elements share concepts with the MANO architecture; however, the characteristics of fog computing solutions lead us to a different approach, since ETSI NFV MANO has been conceived of to be applied in data centers, while in fog environments, each fog node must be able to manage and control its infrastructure. Management and orchestration are still open issues in the fog computing domain due to the dynamic behavior and distributed management of the network, which makes the investigation of using ETSI NFV MANO standards as the base for a fog-based MANO framework implementation necessary.

#### 2.1.5. OMA Lightweight M2M

**Figure 5:**
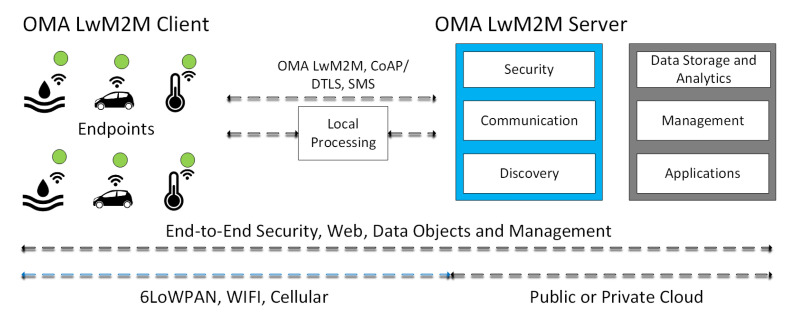
The system architecture of OMA LwM2M.

The Open Mobile Alliance (OMA) designed a device management protocol for sensor networks and M2M environments, which is called Lightweight M2M (LwM2M). LwM2M has been specified by a group of industry experts at the OMAs Device Management Working Group and is based on protocol and security standards from the Internet Engineering Task Force (IETF). OMA LwM2M aims to respond to the demand in the market for a common standard for managing lightweight and low power devices necessary to realize the potential of IoT. The LwM2M protocol builds on an efficient secure data transfer standard called the Constrained Application Protocol (CoAP). The final release has been available since 2014 [13], and the correspondent architecture is shown in Figure 5. As mentioned, the proposed fog computing framework follows ETSI’s oneM2M device management and security standards, which are the main focus of the LwM2M protocol.

#### 2.1.6. TOSCA

In recent years, the Topology and Orchestration Specification for Cloud Applications (TOSCA) [14] has become the standard language for modeling service orchestration in cloud environments in a highly extensible and flexible manner. TOSCA focuses mainly on enhancing the portability and operational management of cloud applications and services across their entire life-cycle, by defining building blocks, requirements and capabilities, which should be taken into consideration in the service orchestration.

#### 2.1.7. Cloudlet

The concept of cloudlet was introduced in [15]. A cloudlet is a small-scale data center that is located at the edges of the network. The main purpose of the cloudlet is to provide cloud resources close to mobile devices as fog computing is currently doing for IoT devices. Cloudlet aims to support resource-intensive mobile applications with latency-sensitive requirements that will emerge in the future 5G network.

#### 2.1.8. Summary

ETSI MEC has been working on a reference architecture for the evolution of the mobile network edges, in order to create a cloud environment close to the RAN, while ETSI MANO has been developing a MANO framework for the provisioning of VNFs and NSs in data centers. Furthermore, ETSI oneM2M has been designing an end-to-end high level architecture for M2M communications, while OMA LwM2M is working on the same path, but focused on device management and security functionalities. Additionally, the cloudlet paradigm is extending the cloud paradigm by placing resources close to mobile devices, while the OpenFog Consortium by using the fog computing paradigm is placing cloud resources at the edges of the network to meet the strict requirements introduced by IoT use cases. Finally, TOSCA is complementing all these standardization efforts by designing a standard language for modeling service orchestration in cloud environments in a highly extensible and flexible manner.

However, all the cited standardization activities still face industry challenges for the full definition of management and orchestration technologies. Most of the discussed reference architectures are still immature and require further investigation and development before they can be operationalized and used by network operators. Research efforts relevant for the specification and implementation of a MANO framework for 5G-enabled smart cities are detailed next.

### 2.2. Research Projects

In recent years, research projects have been carried out to deal with management and orchestration issues in smart cities. The SmartSantander project [16,17] worked on a suitable architectural model for IoT and on the inherent challenges of resource provisioning in smart cities. The SmartSantander framework provides a suitable platform for large-scale experimentation and evaluation of a large set of IoT use cases deployed in several urban scenarios. Furthermore, the CityPulse project [18] has been working on a data analytics framework for smart cities. The CityPulse framework integrates powerful data analytics tools, data aggregation and event detection modules and quality assessment algorithms, which aim to support the development of customized smart city applications. The SusCity project [19] is working on a resilient IoT architecture for smart cities focusing on data collection from multiple sources, in order to develop intelligent management solutions that can help the government and citizens to make appropriate decisions. Furthermore, the VITAL project [20] federates heterogeneous IoT platforms via semantics in a cloud-based environment focusing on smart city scenarios.

Additionally, in [21], a big data network composed of SDN technologies and cloud/fog platforms is presented. Their goal is to reduce the large amount of redundant data and the response time in accessing data services. However, their focus is only on the orchestration of big data services, while our approach is not only concerned with data analytics operations, but also decision making functionalities that can help to autonomously orchestrate smart city applications in a distributed way. Furthermore, their work is based on simulation studies, while our approach is based on an actual deployment within the scope of Antwerp’s City of Things testbed. Furthermore, in a recent article about 5G-enabled smart cities [22], an approach for M2M communication in cognitive 5G networks is presented. This paper introduces a novel decentralized multiple gateway assignment protocol based on multi-channel Carrier Sense Multiple Access (CSMA) for M2M communication in 5G networks. Moreover, a low overhead protocol is also proposed, which increases the throughput of the system and minimizes energy consumption by reducing the message header payload. Simulation studies were carried out to show the effectiveness of the proposed schemes in terms of network lifetime and energy consumption.

Regarding European research, the European Commission (EC), alongside industry manufacturers, telecommunications operators, service providers, SMEs and researchers, created the 5G Infrastructure Public Private Partnership (5G-PPP) to advance the research of 5G technologies in Europe and to build global consensus on 5G networks. The projects supported by the 5G-PPP aim to deliver solutions, architectures, technologies and standards for the ubiquitous next generation communication infrastructures of the coming decade. Regarding management and orchestration, the following 5G-PPP projects should be highlighted. First, the Service Programming and Orchestration for Virtualized Software Networks (SONATA) project [23] aims to deliver an agile service development and orchestration in 5G virtualized networks. SONATA targets both the flexible programmability of software networks and the optimization of their deployments. Secondly, the Coordinated control and spectrum management for 5G heterogeneous radio access networks (COHERENT) project [24] will develop and validate a novel control framework for future mobile networks. The key innovation of COHERENT is the unified programmable control framework to coordinate the underlying heterogeneous mobile networks as a whole. Thirdly, the Self-organized Network Management in Virtualized and Software Defined Networks (SELFNET) project [25] will design and implement an autonomic network management framework to achieve self-organizing capabilities, such as self-protection, self-healing and self-optimization functionalities to deal with major network management problems, which are currently still being manually addressed by network operators, thereby significantly reducing operational costs and improving user experience. Fourthly, the End-to-End Cognitive Network Slicing and Slice Management Framework in Virtualised Multi-Domain, Multi-Tenant 5G Networks (SLICENET) project [26] aims to maximize the potential of the future 5G infrastructures and their services based on advanced software networking and cognitive network management in SDN/NFV-enabled 5G networks. One scenario considered by SLICENET is a smart city use case, the goal of which is to implement a remote water metering and an intelligent public lighting system in the city of Alba Iulia, in Romania. Finally, the Small cEllS coordinAtion for Multi-tenancy and Edge services (SESAME) [27] project’ intention is to develop a new multi-operator-enabled small cell that integrates a virtualized execution platform for deploying VNFs and supporting self-management capabilities.

Although the existing and ongoing research projects cited address some of the requirements of MANO functionalities for 5G networks, they have not yet delivered an integrated and autonomous MANO solution. Therefore, in this article, a fog-based MANO framework is proposed that goes beyond the current state-of-the-art by introducing a fully-integrated and autonomous FN management system, which combines monitoring and data analysis operations alongside management and orchestration decisions for the resource provisioning issue in smart cities.

### 2.3. Open Source Initiatives

This subsection provides a brief summary of the most popular open source activities related to the development of NFV, SDN and M2M technologies.

#### 2.3.1. NFV Open Source Projects

Nowadays, there are several open source projects related to the control and management of VNFs that follow the reference standards provided by ETSI. In Table 1, the most popular NFV open source projects today are shown.

#### 2.3.2. SDN Open Source Projects

With the growing development of NFV and SDN technologies, the number of open source projects related to SDN controllers is rapidly increasing. In Table 2, the working groups related to SDN controllers are shown.

#### 2.3.3. M2M Open Source Projects

Currently, with the growing amount of connected devices, the number of open source initiatives related with M2M management issues is rapidly increasing. In Table 3, the most popular M2M standardization efforts are shown.

### 2.4. Ambition

In this article, a fog computing framework is proposed to enable autonomous MANO functionalities for smart city applications in 5G Networks. This work extends the current state-of-the-art within fog computing, NFV and MANO paradigms by introducing a fully-integrated and autonomous fog node management system, which combines monitoring and data analysis operations alongside management and orchestration decisions. Our approach follows the guidelines of the ETSI NFV MANO architecture and the standards of ETSI oneM2M in terms of device management and security. Furthermore, a novel fog protocol is introduced, which enables the exchange of application service information between FNs and the cloud layer, improving the performance of application-to-resource provisioning results.

In summary, this work looks beyond existing and ongoing research projects that individually address some of the requirements, but have not yet delivered an integrated and autonomous solution for the resource management and orchestration issue in smart cities. Smart city applications will introduce a set of stringent requirements, such as low latency and high mobility, since services can be requested on-demand simultaneously by multiple devices on different locations. To deal with these limitations, efficient resource provisioning is needed in order to address these constraints introduced by smart city applications while minimizing resource costs. Therefore, in this article, a fog-based management and orchestration framework is proposed to deal with the application service placement problem in smart cities. The framework is presented in the next section.

## 3. A Fog-Based Management and Orchestration Framework for 5G Smart Cities

In this section, a fog computing framework is presented for the management and orchestration of smart city applications. First, architectural challenges introduced by the fog computing paradigm are presented. Then, a system overview of our proposed framework is detailed. Furthermore, a fog protocol is introduced to provide exchange of application service provisioning information between multiple FNs. Finally, a hierarchical and distributed data monitoring and analysis approach based on our framework is detailed.

### 3.1. Challenges of a Fog-Based MANO Architecture

To deal with diverse IoT requirements, such as latency, energy efficiency and mobility, the fog computing paradigm has been introduced. Centralized cloud solutions are not suitable for future IoT applications, with real-time constraints and enormous volumes of data to be transported in the network. This way, fog computing architectures can provide effective ways to overcome many limitations of the existing network architectures that rely only on computing resources in the cloud and on end-user devices.

#### 3.1.1. Latency Constraints

Fog computing will allow real-time processing and data analytics at the edges of the network, which will enable the deployment of delay-sensitive applications and the control of time-sensitive network functionalities close to end devices. This will allow coping with the strict requirements of the future smart city applications. This way, fog computing will help to reduce the service provisioning delay for most applications.

#### 3.1.2. Security Challenges

Existing security solutions are designed for protecting enterprise networks and data centers by providing perimeter-based protections. These security services are no longer adequate for addressing the new security challenges in the emerging IoT systems. Fog computing architectures will enable a wide range of security functions, such as distributed malware monitoring for the multiple IoT devices to compensate these devices’ limited security and primarily take advantage of the gathering of local information in order to detect threats and attacks in a timely manner.

#### 3.1.3. Network Bandwidth Constraints

Centralized solutions are not suitable for smart city scenarios since sending all the data collected by IoT devices to the cloud layer will require an enormous amount of network bandwidth, which centralized solutions and Low Power Wide Area Network (LPWAN) technologies cannot support. Fog computing enables data processing and analytics operations locally, which drastically reduces the amount of data that needs to be sent to the cloud layer.

#### 3.1.4. Resource-Constrained IoT Devices

IoT devices have limited resources (battery, computational power, storage and memory capacity). It is not feasible to rely only on these devices to fulfill all the needed computational operations. FNs will carry out most of the computational tasks on behalf of resource-constrained IoT devices, hence reducing these devices’ complexity, deployment costs and energy consumption.

#### 3.1.5. High Dynamicity

Mobility is an important requirement of IoT scenarios since resources can be requested on-demand simultaneously by multiple devices at different locations. Furthermore, IoT introduces serious challenges on how to address seamless mobility in heterogeneous environments, since devices are constantly moving and accessing the medium through different technologies. To deal with these inherent mobility challenges, the fog computing paradigm has been introduced to provide resources and services on the edges of the network to effectively handle handover procedures.

#### 3.1.6. Distributed Data Analysis

As IoT devices can send their data samples to local FNs, monitoring and anomaly detection operations can be performed in a distributed way. If unusual events or abnormal behaviors are detected in the data, faster response times can be achieved. This way, malfunctions in IoT devices can be detected and transmissions of incorrect information can be avoided in a timely manner, which can improve the network reliability.

#### 3.1.7. Local Autonomous Operations

By enabling decisions and autonomous operations locally, it is possible to reduce the amount of data that needs to be sent to the cloud layer, decreasing the latency in the communication and improving the response time in case network failures are detected.

### 3.2. System Architecture Overview

Our approach follows the guidelines of the ETSI NFV MANO architecture for infrastructure management and orchestration, defined in the context of NFV technologies, extending it with additional software components, which will offer not only high computing performance and intelligence in 5G smart cities, but also monitoring and data analysis functionalities to provide secure and reliable communications. Moreover, our solution incorporates the standards of ETSI oneM2M regarding device management and security.

#### 3.2.1. Global Overview of the Fog-Based MANO Framework

**Figure 6:**
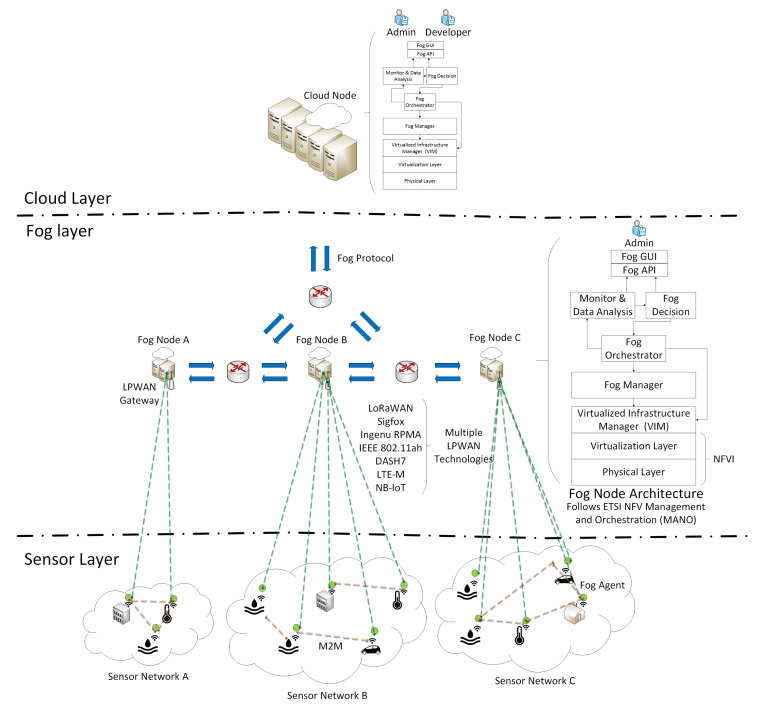
Overview of the proposed fog-based MANO framework for 5G-enabled smart cities.

Figure 6 provides an overview of our proposed fog computing framework. IoT devices, mainly sensors and actuators, communicate with LPWAN gateways, which are linked with the fog layer through multiple FNs. Each FN is an autonomous system managing a given set of computational resources. FNs communicate with the cloud layer through a Cloud Node (CN), which is the top level management entity. The CN is responsible for the global management and control operations in the network. FNs will be able to communicate with other FNs and with the CN based on an application layer P2P fog protocol, which enables the exchange of application service provisioning information between fog nodes and the cloud layer, improving the decision making process related to the resource provisioning in 5G smart cities.

#### 3.2.2. Fog Node System Architecture

**Figure 7:**
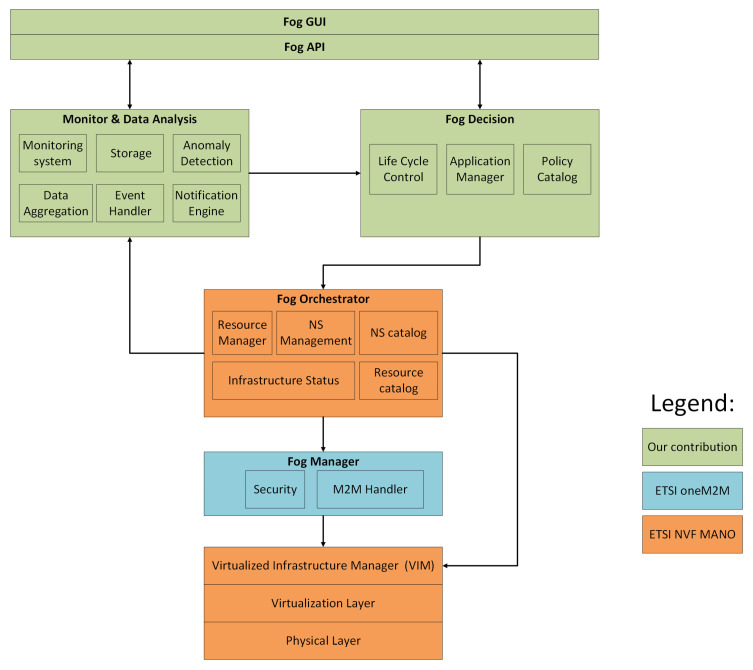
Detailed overview of the FN architecture.

Figure 7 presents the detailed architecture of the FN autonomous management system. Some FN architectural components share concepts with the ETSI NFV MANO architecture; however, the nature and behavior of the FNs in the infrastructure lead us to a different approach, since ETSI NFV MANO has been conceived to be applied in data centers, and our proposal is aligned with fog computing architectures. The FN must setup and manage its infrastructure and associated devices in an autonomous manner. Therefore, each FN is managing a set of computational resources by using a virtualization layer residing over a physical layer, offering virtualization of the main network functionalities. The VIM performs the life-cycle management of the multiple network functions deployed on the network.

The next upper software module, is the Fog Manager (FM) component, which is responsible for managing the attached IoT devices through a Fog Agent (FA). The FM addresses the device management and M2M security guidelines defined by ETSI oneM2M. The FM module is mainly responsible for:

- Device discovery operations;
- Updating the devices’ configurations through the FA;
- Keeping track of the devices’ mobility;
- The security in M2M communications;

Furthermore, each FN has its own instance of a Fog Orchestrator (FO) component. The FO module is mainly responsible for the following operations:

- Life-cycle management of micro-services (including instantiation, scale-out/in, termination);
- Interface with the monitoring and data analysis system;
- Interface with the Fog Decision (FD) module;
- Responsible for the registration of new VNFs and NSs;

On the one hand, a monitor and data analysis component is responsible for gathering information about the current state of the computational resources, such as CPU, memory and storage and collecting data samples from the different IoT sensors through the M2M handler. This module is composed of databases of measurements, events, warnings and notifications, which will help to identify network failures, policy violations and security threats on the network in a timely manner. In fact, this component keeps an updated status of the overall FN system alongside the connected IoT devices. On the other hand, our FN system architecture foresees a fog decision module, which is the component housing the intelligence, mainly responsible for:

- Life-cycle control and management decisions;
- Self-configuration functionalities;
- Applying the network behavior desired by network administrators;
- Providing autonomous responses to unknown situations detected by the monitoring and data analysis module;

The FD module will use different sets of machine learning algorithms to provide self-management responses to unusual events or malfunctions that can be detected in the network. The FD module will apply network strategies indicated in the policy catalog. Moreover, application and service migration requests will be handled by the FD module. The main outcome of the FD module is a set of actions that need to be deployed in the network, which will be done by the FO.

Each FN is composed by a northbound Application Programming Interface (API) defining a standardized entry point to the FN autonomous system. This interface defines the interaction between network administrators and FNs. This API will provide access to all configuration, management and reporting capabilities provided by FNs.

Finally, each FN also provides a Fog Graphical User Interface (GUI), where network administrators can interact and configure each FN. Furthermore, network administrators can obtain a broader view of the behavior of the network, in terms of resource usage, application service deployments, etc., and present this status in a command and control center. Both the fog GUI and fog API will provide the necessary tools for network administrators to stop or manually enforce any kind of action on an FN despite the fact that each FN is executing a significant part of the processes in an autonomous way.

In summary, FNs are fully-integrated and autonomous management systems with data analysis and decision making functionalities. Essentially, an FN manages a small set of computational resources such as in small cloud environments. This way, an FN can be considered a small cloud entity. Our goal is to distribute these entities across the network in order to provide autonomous distributed operations and decisions in future 5G smart cities.

#### 3.2.3. Cloud Node System Architecture

The architecture of the CN is essentially the same of the FN. However, CN functionalities are different since the CN is responsible for the global management and control operations in the network. The CN is mainly responsible for the following operations:

- Software updates of VNFs or NSs on the FNs;
- Coordination and control of FNs;
- Global data analysis operations;
- Overall QoS and Service Level Agreements (SLAs) monitoring;
- Applying the global network behavior indicated by network administrators;

### 3.3. Fog Protocol: Enabling Exchange of Application Service Information between Fog Nodes and the Cloud Layer

In the presented framework, each FN will communicate with the other FNs and with the CN in the cloud layer in order to exchange resource provisioning information. To the best of our knowledge, no suitable way to provide the exchange of this kind of information between the fog and the cloud layer is available in literature. This is still a key research challenge in the fog computing domain. By taking into account the main advantages of fog computing architectures, which are minimized latency and reduced bandwidth use, a suitable fog protocol should be lightweight, transport agnostic and customizable, since QoS parameters must be selected according to the requested application. This way, the fog protocol should be able to cope with the requirements introduced by the different IoT use cases, such as low-power communication devices, low bandwidth data links, low latency communications and high mobility scenarios.

The allocation of smart city application composed of micro-services with delay-sensitive requirements must be reactive if the device that is requesting the application is moving through the network area. This will imply a need for fast service migrations, which can be achieved if messages are exchanged between the multiple FNs with information regarding services and applications. This way, each FD module can decide where and when it is more suitable to deploy and instantiate each instance of the micro-services composing the smart city application since the application service topology information of the network will be known by the different FNs. Therefore, in the presented framework, an application layer fog protocol based on a lightweight version of the OSPF [37] routing protocol for the exchange of application service information between FNs and the cloud layer is proposed. OSPF provides routing functionality within the same domain. It was designed to optimize the propagation of topological changes in the network. Our goal is to use a lightweight application layer protocol based on the advantages of OSPF to propagate the application service logic information in the network, such as micro-service allocations and migrations, so that each FN knows exactly the application service topology, i.e., what and where each micro-service is allocated. This way, application service information can be shared among FNs similar to P2P networks. In fact, the foreseen fog protocol is a hierarchical application layer P2P networking protocol based on the advantages of OSPF.

**Figure 8:**
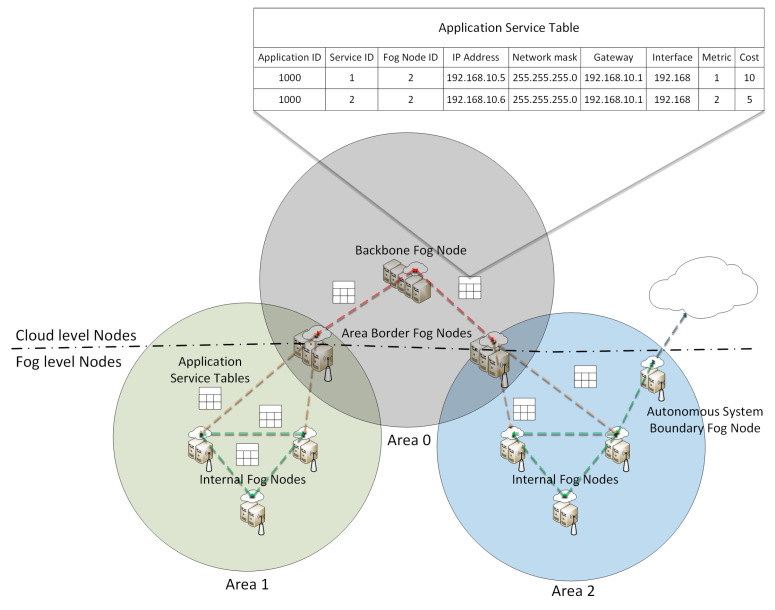
P2P fog protocol: based on a lightweight version of OSPF to exchange application service provisioning information between fog nodes and the cloud layer.

In Figure 8, a topology scenario of our fog protocol is presented. By using a fog protocol based on the advantages of OSPF, it will be possible to achieve rapid decisions related to the resource provisioning in smart cities, based on the exchange of application service tables. Each instance of applications and services have a unique ID allowing one to know exactly which instance is allocated to a certain FN. Each FN has an Internet Protocol (IP) address, IPv4 or IPv6, and also a unique ID. These attributes are transmitted as headers on the application service tables. These application service tables enable the exchange of resource operational information allowing the FD module to provide fast decisions on the resource provisioning because the overall network service allocation will be known by each fog node system, and therefore, decisions on service migrations can be achieved in a timely manner.

The fog protocol will split the network domain into multiple areas as in OSPF. FNs will only need to know the application service logic of the areas to which they are connected. Furthermore, FNs are classified according to the functions they will perform. As in OSPF, internal FNs are those connected to networks belonging to the same area, while Area Border Fog Nodes (ABFNs) are those connected to networks belonging to multiple adjacent areas. ABFNs will summarize the application service logic information of their attached areas for distribution through the backbone area, the cloud layer. Backbone FNs are those connected to the backbone area, including ABFNs. Autonomous System Boundary Fog Nodes (ASBFNs) are those that exchange application service information with FNs belonging to other service provider domains.

### 3.4. Distributed Data Monitoring and Analysis for 5G Smart Cities

**Figure 9:**
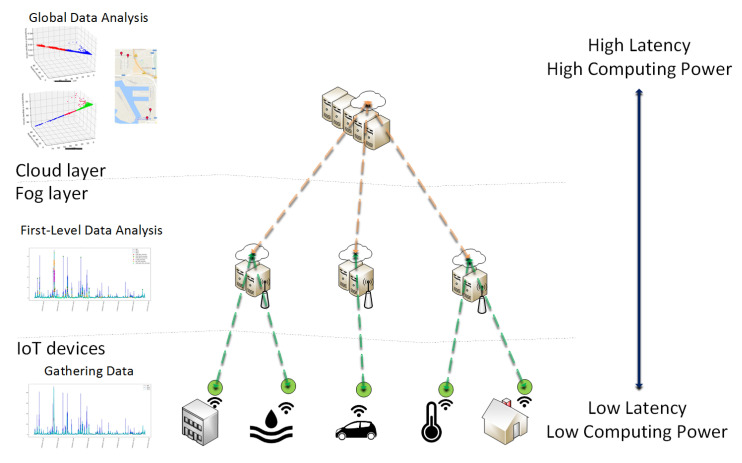
Overview of the distributed data analysis approach for 5G smart cities.

The future 5G network architecture will support the integration of a massive number of infrastructure components and application services in smart cities. Regarding 5G-enabled applications, one of the remaining challenges is how to provide efficient resource allocation operations, because of the stringent requirements introduced by these type of applications: extremely low latencies, ultra-reliable communications and massive M2M communications. For smart city applications, services can be placed in a highly congested area, which would result in a higher latency in the communication between the fog nodes and the IoT device. This is unfeasible for delay-sensitive applications, since these require very low latencies, meaning these applications must be allocated on fog nodes close to the IoT sensor enabling the control of time-sensitive network functionalities close to the device. Furthermore, to provide a secure and reliable communication, it is necessary to perform data processing and analysis operations in a distributed way in order to detect anomalous behaviors and abnormal events in a timely manner so that appropriate actions can be performed in real time. In traditional centralized cloud solutions, all data are sent to the cloud layer, and then, monitoring and analysis operations are executed, which is not suitable for IoT scenarios. As mentioned before, in the proposed architecture, a monitoring and analysis component is present in the architecture of the FN. The proposed monitoring and data analysis approach is shown in Figure 9. This way, data will be processed near the end devices at the edge providing low-latency and scalable anomaly detection solutions, which will provide appropriate responses to protect infrastructure components, as well as application-level communication. First, IoT sensors will be distributed at various public infrastructures to monitor their condition variations over time. Then, these sensors will forward the raw data into the fog layer, where each FN will process the data samples associated with a local group of sensors and perform data analysis operations in a timely manner.

The outcomes of this first-level analysis will be processed by an event handler and a notification engine. If anomalous events are already detected on the data, notifications and alerts are generated and sent to the cloud layer, the CN and also to the IoT devices. Meanwhile, the unusual data samples that generate the alarms are also reported to the cloud layer in order to perform global behavior analysis based on machine learning techniques that require a higher computational power. Therefore, the CN will provide a broader view of the behavior of the network. Moreover, in-depth pattern recognition and event detection operations can be performed at the CN in order to support the FD module to apply more appropriate reactive and proactive responses.

### 3.5. Summary

The framework presented in this section enables autonomous MANO functionalities for smart city applications in diverse IoT scenarios. First, architectural challenges of the fog computing paradigm have been presented. Then, a system overview of our proposed framework has been detailed. Our work provides MANO capabilities to 5G networks, which according to our knowledge are still not yet fully explored in literature since previous and ongoing research has not yet delivered an integrated and autonomous solution for the resource management and orchestration issue in smart cities. Our approach follows the guidelines of the ETSI NFV MANO architecture and the standards of ETSI oneM2M regarding device management and security aspects, by extending it with additional software components that will offer not only high computing performance, but also monitoring and data analysis functionalities to provide secure and reliable communications in smart cities. Then, a novel application layer P2P fog protocol based on the advantages of the OSPF routing protocol has been introduced to provide exchange of application service provisioning information between the multiple FNs in the fog layer and the CN in the cloud layer. Instead of forwarding routing tables across the network, the objective of the foreseen fog protocol is to forward application service information, such as micro-service allocations and migration requests, enabling fast decisions related to the resource provisioning in smart cities. Finally, a hierarchical and distributed data monitoring and analysis approach based on our fog computing framework has been detailed. The proposed framework enables low-latency and scalable data analysis operations in order to detect abnormal events in a timely manner to apply appropriate actions in real time. In the next section, an anomaly detection use case is presented, which has been used to validate our distributed data analysis approach in 5G smart cities for the proposed fog-based MANO framework.

## 4. Evaluation Use Case

In this section, the evaluation scenario is introduced. Then, the datasets are presented. Finally, the data analysis operations used in the evaluation are described.

### 4.1. Scenario Description: Air Monitoring Application

The evaluation scenario is based on a use case within the scope of Antwerp’s City of Things testbed [38]. The objective of our air monitoring application is to show the current status of the environment in the City of Antwerp and alert citizens of ambient pollution through a notification system in near real time. Regarding the current literature in air monitoring applications for smart cities, the proposed approach in [39] must be highlighted. In the article, a novel cloud-based approach for air quality monitoring is discussed. The approach is based on the design of two sensor front ends: a stationary air quality sensor that connects to the cloud via Ethernet and General Packet Radio Service (GPRS) and a portable sensor that connects to the smartphone via Bluetooth 4.0.

In contrast, our approach is based on the deployment of a set of air quality sensors mounted on the roofs of bpost’s (Belgian postal services) delivery cars as an initial proof of concept. These sensors send measures of typical gases and climate data, such as temperature and humidity, which are then annotated with GPS locations. Furthermore, these sensors allow gathering real-time air quality information with broad city coverage, since each car is continuously driving around in the city.

### 4.2. Datasets

**Figure 10:**
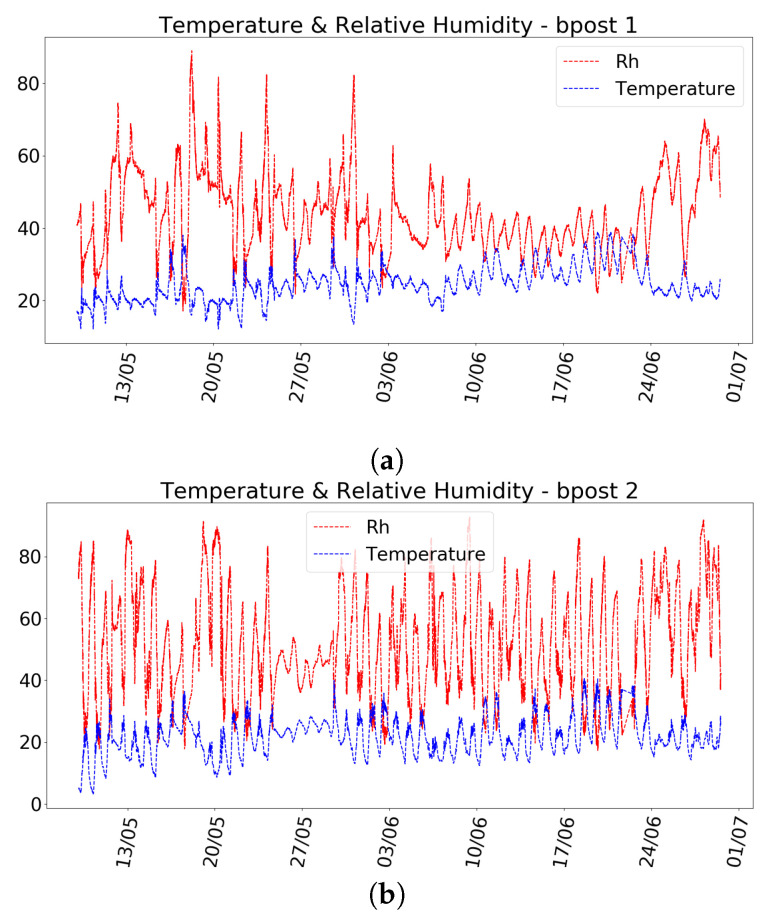
Temperature and relative humidity values collected from two bpost cars. (**a**) bpost Car 1; (**b**) bpost Car 2.

A summary of the characteristics of the datasets gathered for the evaluation is shown in Table 4. The two datasets come from two different bpost cars and consist of Particle Matter indicators (PM1, PM2.5 and PM10), temperature and humidity values that are annotated with a GPS location. The datasets have been collected by our research group between 9 May 2017 and 29 June 2017. Temperature and relative humidity values collected by bpost Car 1 and bpost Car 2 are shown in Figure 10a,b, respectively.

### 4.3. Data Analysis Operations

In this subsection, the data analysis operations used in the evaluation are introduced.

#### 4.3.1. First-Level Analysis: Unsupervised Outlier Detection Algorithms

Outlier detection is related to the identification of unusual data samples when compared to the rest of the dataset. The Robust Covariance (RC) and the Isolation Forrest (IF) algorithms have been employed as a first-level analysis to be performed by FNs in the fog layer. These algorithms have been evaluated by using Scikit-Learn [40], a machine learning library written in Python.

#### 4.3.2. Global Analysis: GPS Locations of Outliers

The unusual measurements detected by FNs through outlier detection must be further analyzed by application experts in order to extract more information from them. As such, FNs will trigger appropriate alarms and notifications, which will be forwarded through the network to the CN. Then, these unusual samples will be sent to the CN so that more complex and resource-consuming operations can be performed, e.g., for root cause analysis. In our evaluation, the outliers have been compared with the annotated GPS locations as a global analysis operation.

### 4.4. Fog-Cloud Infrastructure Dimensioning

Nowadays, low power wireless technologies and fog computing architectures have gained tremendous emphasis due to the massive growth of connected devices in the network. The need for connecting simple IoT devices, such as sensors and actuators, is increasing rapidly. Variables used in our fog-cloud infrastructure dimensioning are shown in Table 5.

In fog computing architectures, fog nodes are usually located within one hop from the IoT sensors. The variable *C* is used to indicate the communication range in meters between a fog node and an IoT sensor. Two variables, $U_{LPWAN}$ and $D_{LPWAN}$, are used to indicate the upload and the download data rate of the LPWAN technology, respectively. Then, the number of bits in each data sample is given by *N*. The number of data samples to be transmitted is given by *R*. This way, the upload and the download transmission time of a packet between an IoT sensor and a fog node can be expressed as shown in (1) and in (2), respectively.

(1) $$T\left(U_{LPWAN}\right)=\frac{N\times R}{U}$$

(2) $$T\left(D_{LPWAN}\right)=\frac{N\times R}{D}$$

Moreover, the communication between a fog node and the cloud node is via the Internet, where the upload speed is given by $U_{Fog}$ and the download speed is given by $D_{Fog}$.

## 5. Evaluation Results

### 5.1. Unsupervised Outlier Detection

**Figure 11:**
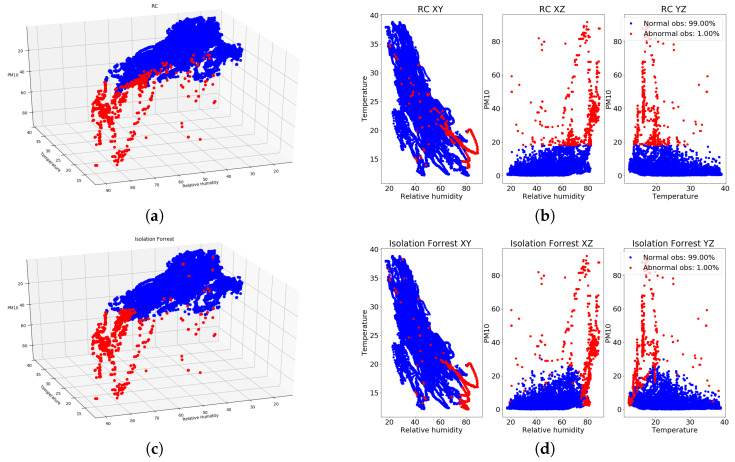
Outcomes of RC and IF algorithms (blue color: normal samples, red color: abnormal samples) for PM10, temperature and relative humidity values collected by the bpost Car 1. (**a**) RC with 1.0 (3D perspective), bpost Car 1; (**b**) RC with 1.0% (3D planes), bpost Car 1; (**c**) IF with 1.0% (3D perspective), bpost Car 1; (**d**) IF with 1.0% (3D planes) bpost Car 1.

**Figure 12:**
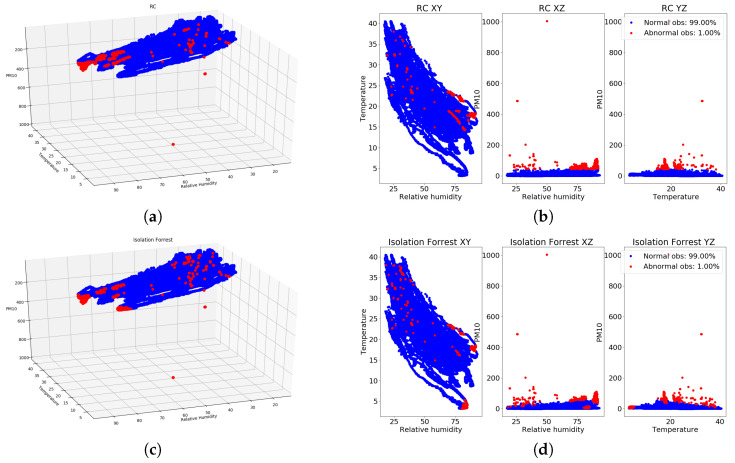
Outcomes of RC and IF algorithms (blue color: normal samples, red color: abnormal samples) for PM10, temperature and relative humidity values collected by the bpost Car 2. (**a**) RC with 1.0% (3D perspective), bpost Car 2; (**b**) RC with 1.0% (3D planes), bpost Car 2; (**c**) IF with 1.0% (3D perspective), bpost Car 2; (**d**) IF with 1.0% (3D planes), bpost Car 2.

In Figure 11a,b, the outcomes of the RC algorithm for the three dimensions regarding PM10, temperature and relative humidity for the bpost Car 1 are shown, with a contamination of 1.0% indicating that the RC algorithm intends to find the 1.0% of samples, which can be considered as abnormal. Similarly, in Figure 11c,d, the results obtained for the IF outlier detection algorithm with a contamination of 1.0% for the bpost Car 1 are shown. In addition, in Figure 12a,b, the outcomes of the RC algorithm for the bpost Car 2 are presented, while in Figure 12c,d, the results obtained from the IF algorithm are shown. As can be observed, clear similarities are present in the results obtained by both algorithms for the two bpost cars. PM10 values above 30 ppm collected by both cars are marked as outliers by both algorithms, which indicates that these samples can be considered as unusual measurements.

### 5.2. Global Analysis: GPS Locations of Outliers

**Figure 13:**
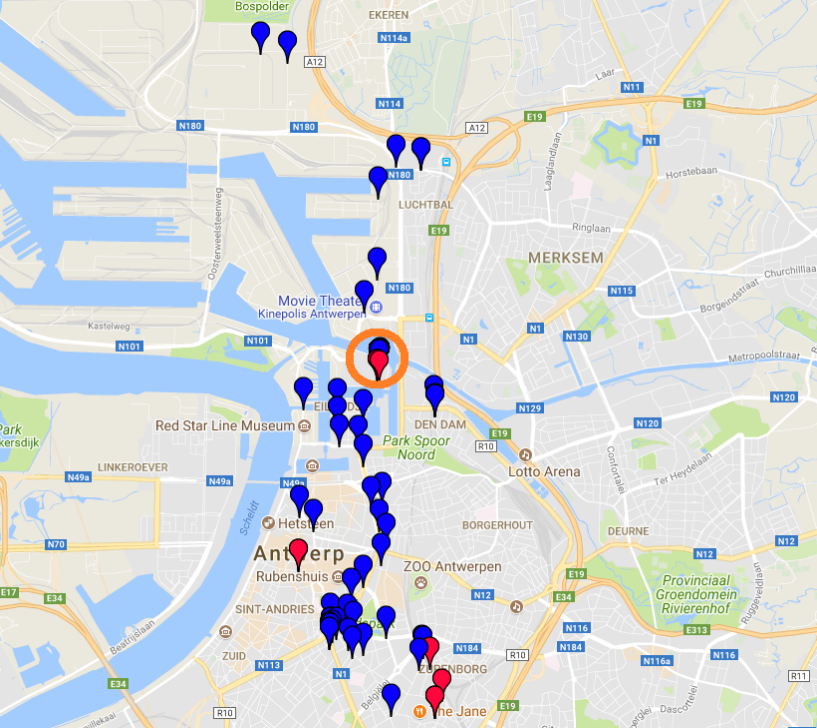
GPS locations collected by the bpost cars (bpost1, red/bpost2, blue). These values have been marked as outliers by both outlier detection algorithms considered in the evaluation.

As previously mentioned, as a global analysis operation, the outliers detected by the RC and IF algorithms have been linked with the GPS locations available in the datasets. Only measurements marked as outliers by both algorithms have been considered. In Figure 13, the GPS locations where PM10 values above 70 ppm have been collected by the bpost cars, which have been marked as outliers by both RC and IF outlier detection algorithms, are shown on a map of the city. Regarding bpost Car 1 measurements, most of the data samples marked as outliers have been collected in the warehouse (area highlighted in orange in Figure 13), where the bpost cars usually stay at night. These high values of PM10 can be related to organic compounds, which were inside the warehouse at the time of the measurements. On the other hand, the outliers measured by bpost Car 2 have been collected across the city of Antwerp. These values can be related to high traffic volumes in the city at the time of the measurements. Furthermore, a large number of samples collected by bpost Car 2 have been marked as outliers, when the car stayed in the warehouse. These outliers can be explained by the high relative humidity and the high values of the PM10 indicator.

Global analysis operations should be conducted in the cloud layer so that unusual measurements can be understood. This way, citizens and government agencies can receive live input of the city traffic, which should be used to temporarily modify traffic rules, e.g., reduce the maximum driving speed on certain highways. Furthermore, in the long term, these global operations can provide an effective way on how city policies should be changed.

### 5.3. Fog-Cloud Infrastructure Analysis

In the fog-cloud infrastructure evaluation, the communication between a fog node and the cloud node is via the Internet, where the upload speed is 5 Mbps and the download speed is 2.5 Mbps. Furthermore, a 500-m communication range has been considered between a fog node and an IoT sensor. The communication between a fog node and an IoT sensor is performed by a LPWAN technology. An IEEE 802.11ah [41] wireless network has been considered as the LPWAN technology, because it is one of the most promising LPWAN technologies with very high data rates. High data rates are an important requirement of the evaluation use case, because timely alerts need to be sent to the IoT sensor if a malfunction is already detected on a fog node. This way, an upload data rate of 2 Mbps and a download data rate of 1 Mbps have been considered based on IEEE 802.11ah. Although the maximum throughput of IEEE 802.11ah is higher, with this lower limitation, it has been assumed that good channel conditions are always achieved and that all IoT sensors can communicate at these data rates. Other LPWAN technologies, such as LoRaWAN [42] and Sigfox [43], have not been considered because very low data rates and duty cycle restrictions make it impossible for these LPWAN technologies to send a data sample every minute.

Considering that for our use case, each upload message is composed of a string of 12 chars (GPS location, geohash) equal to 12 bytes, a 32 bit integer (timestamp) equal to 4 bytes and 5 floating point 64-bit numbers (particle matter indicators, temperature and humidity) equal to 40 bytes, the total number of payload bytes to be transmitted per minute from the IoT sensor to the fog node is 56 bytes. On the other hand, each download message to be transmitted from the fog node to the IoT sensor in case of unusual behavior or malfunction is composed of a string of 12 chars (GPS location, geohash) equal to 12 bytes and a byte defined by 3 alarm bits and 5 bits for 32 types of predefined messages. Therefore, each upload message is transmitted with at least 56 bytes, which is equal to 448 bits and each download message with 13 bytes, which is equal to 104 bits. Moreover, the fog node will send 1% of the total data samples to the cloud node for global analysis operations. However, in a traditional centralized cloud solution, all data samples need to be transported from the IoT sensor to the cloud node. Based on these assumptions, our fog-based framework has been evaluated and compared with a traditional centralized cloud solution in terms of response time in case abnormal samples are detected and in terms of network bandwidth usage. The comparison is presented in Table 6.

Our results show that to send all data samples to the CN, 6.27 seconds are needed, while in our fog computing approach, only 14.56 milliseconds are required since only 1% of the data is transmitted to the cloud node. Moreover, our proposal only requires 1.46% of the available bandwidth between the fog node and the cloud node, while in a traditional centralized solution, on average, six-times the available bandwidth is needed (627.2%) only for a single fog node to transmit all data samples from only one IoT sensor, which makes it impossible for centralized cloud solutions to comply with smart city use cases. The end-to-end delay in case an abnormal sample is detected is lower in our solution since the first-level analysis is executed on fog nodes. On average, our solution needs 0.33 milliseconds to send an alarm or notification to the IoT sensor, while a traditional approach requires 0.46 milliseconds, but with an enormous amount of network bandwidth.

## 6. Conclusions

In this article, a fog computing framework for the management and orchestration of smart city applications in 5G wireless networks is presented. Fog computing has been introduced to deal with the growing amount of connected devices in the upcoming years, by placing computational resources on the edges of the network. This way, fog computing solutions can provide effective ways to overcome the strict requirements introduced by IoT scenarios, such as low latency, high energy efficiency and high mobility. The proposed framework extends the current state-of-the-art within fog computing, NFV and MANO paradigms by providing autonomous capabilities for the resource provisioning in 5G-enabled smart cities. The main contribution of our work is its fully-integrated and autonomous fog node management system with data analysis and decision making functionalities alongside a foreseen novel application layer P2P fog protocol based on the OSPF routing protocol enabling the exchange of application service provisioning information between fog nodes and the cloud layer. Moreover, an anomaly detection use case has been evaluated focusing on the proposed framework. The evaluation results show that the presented approach achieves a substantial reduction in terms of network bandwidth usage when compared with traditional centralized cloud solutions. Our approach only requires 1.46% of the available network bandwidth between a fog node and a cloud node. Moreover, the proposed framework can send timely alerts to IoT sensors in case abnormal samples are detected, since a first-level analysis is performed in fog nodes. As future work, prototypes and proof-of-concepts of our fog computing framework will be implemented. Moreover, simulation studies will be carried out to evaluate the operational aspects of the foreseen fog protocol.

## Figures and Tables

**Table 1:** NFV open source projects.

Project	Description
OpenStack [28]	OpenStack allows the management of virtualized resources such as virtual disks, computational nodes and networks. OpenStack addresses the management paradigm of the ETSI MANO NFV architecture.
OpenBaton [29]	OpenBaton is an ETSI NFV compliant NFVO. OpenBaton has a modular implementation allowing the integration of other software modules without having to modify or understand its core implementation. OpenBaton supports a plugin system to incorporate virtual infrastructure managers, such as Openstack.
OpenMano [30]	OpenMANO is an approach to the management and orchestration of ETSI NFV standardization. However, although OpenMano allows the definition of network services and their instantiation and deletion, it does not provide a virtual network function manager.

**Table 2:** SDN open source projects.

Project	Description
OpenDayLight [31]	OpenDayLight is an open source project hosted by The Linux Foundation working on a platform to accelerate the adoption of SDN technologies. OpenDayLight aims to solve a wide range of common network problems in an automatic manner by making networks more programmable and intelligent.
ONOS [32]	ONOS is an open source SDN networking operating system that aims to provide a scalable and modular platform that eases the development of SDN applications and services.
Ryu [33]	Ryu is a component-based SDN framework. Ryu provides software components with well defined APIs to create new network management and control applications.

**Table 3:** M2M open source projects.

Project	Description
OMA LwM2M [34]	OMA LwM2M is a device management protocol designed for IoT networks and the demands of M2M environments.
Leshan [35]	Leshan is a LwM2M server and client Java implementation, which provides software libraries to help the development of other LwM2M applications.
5G-EmPOWER [36]	5G-EmPOWER is a multi-access edge computing operating system supporting heterogeneous radio access technologies. 5G-EmPOWER aims to provide full visibility of the network state and allow the dynamic deployment and orchestration of NSs.

**Table 4:** Evaluation datasets. bpost, Belgian postal services.

Dataset Name	No. of Records	Description
bpost 1	70636	Particle matter indicators (PM1, PM2.5, PM10), Temperature, Humidity and GPS locations from bpost car 1 between 9 May 2017 and 29 June 2017
bpost 2	70640	Particle matter indicators (PM1, PM2.5, PM10), Temperature, Humidity and GPS locations from bpost car 2 between 9 May 2017 and 29 June 2017

**Table 5:** Variables of the fog-cloud infrastructure dimensioning.

Symbol	Description
*C*	Communication range in meters.
$U_{LPWAN}$	Upload data rate of the LPWAN technology in Mbps.
$D_{LPWAN}$	Download data rate of the LPWAN technology in Mbps.
$U_{Fog}$	Upload speed between a fog node and the cloud node in Mbps.
$D_{Fog}$	Download speed between a fog node and the cloud node in Mbps.
*R*	Number of data samples to be transmitted.
*N*	Number of bits in each data sample.
*T*	Transmission time of a packet.

**Table 6:** Performance evaluation of our fog-cloud infrastructure.

Variable	Fog-Cloud	Centralized Cloud
*C*	500 m	
$N_{Upload}$	448 bits	
$N_{Download}$	104 bits	
$U_{LPWAN}$	2 Mbps	
$D_{LPWAN}$	1 Mbps	
$T_{Upload:Sensor-Fog}$	0.224 ms	
$T_{Download:Sensor-Fog}$	0.104 ms	
$U_{Fog}$	5 Mbps	
$D_{Fog}$	2.5 Mbps	
$T_{Upload:Fog-Cloud}$	0.090 ms	
$T_{Download:Fog-Cloud}$	0.041 ms	
$R_{Sensor-Fog}$	70,000 data samples	
$R_{Fog-Cloud}$	700 data samples (1%)	70,000 data samples (100%)
$N_{Fog-Cloud}$	72.8 Kbps	31.36 Mbps
Transporting Data Samples to the Cloud	14.56 ms	6.27 s
Network Bandwidth Usage (%)	1.46%	627.2%
End-to-End delay for Alert/Notification	0.33 ms	0.46 ms

## Abbreviations

The following abbreviations are used in this manuscript:

5G-PPP	5G Infrastructure Public Private Partnership
AE	Application Entity
API	Application Programming Interface
BSS	Business Support System
CN	Cloud Node
CoAP	Constrained Application Protocol
COHERENT	Coordinated control and spectrum management for 5G heterogeneous radio access networks
CSE	Common Service Entity
CSMA	Carrier Sense Multiple Access
D2D	Device-to-Device
EC	European Commission
EMS	Elemental Management System
ETSI	European Telecommunications Standards Institute
FA	Fog Agent
FD	Fog Decision
FM	Fog Manager
FN	Fog node
FO	Fog Orchestrator
GPRS	General Packet Radio Service
GUI	Graphical User Interface
ID	Identifier
IETF	Internet Engineering Task Force
IF	Isolation Forrest
IoT	Internet of Things
IP	Internet Protocol
LPWAN	Low Power Wide Area Networks
LwM2M	Lightweight M2M
M2M	Machine-to-Machine
MANO	Management and Orchestration
ME	Mobile Edge
MEC	Mobile Edge Computing
MNO	Mobile Network Operator
NFV	Network Function Virtualization
NFVI	Network Function Virtualization Infrastructure
NFVO	Network Function Virtualization Orchestrator
NE	Network Service
OMA	Open Mobile Alliance
OSS	Operations Support System
OSPF	Open Shortest Path First
P2P	Peer-to-Peer
QoS	Quality of Service
RAN	Radio Access Network
RC	Robust Covariance
REST	Representational State Transfer
SDN	Software-Defined Networking
SESAME	Small cEllS coordinAtion for Multi-tenancy and Edge services
SLA	Service Level Agreements
SOA	Service-Oriented Architecture
SONATA	Service Programming and Orchestration for Virtualized Software Networks
TOSCA	Topology and Orchestration Specification for Cloud Applications
VIM	Virtualized Infrastructure Manager
VNF	Virtual Network Function
VNFM	Virtualized Network Function Manager

## References

[1] Cisco Visual Networking Index: Global Mobile Data Traffic Forecast Update, 2016–2021 White Paper. http://www.cisco.com/c/en/us/solutions/collateral/service-provider/visual-networking-index-vni/mobile-white-paper-c11-520862.pdf.

[2] Gupta A., Jha R.K. (2015). A survey of 5G network: Architecture and emerging technologies. IEEE Access.

[3] Vaquero L.M., Rodero-Merino L. (2014). Finding your way in the fog: Towards a comprehensive definition of fog computing. ACM SIGCOMM Comput. Commun. Rev..

[4] Kobo H.I., Abu-Mahfouz A.M., Hancke G.P. (2017). A Survey on Software-Defined Wireless Sensor Networks: Challenges and Design Requirements. IEEE Access.

[5] Chiang M., Zhang T. (2016). Fog and IoT: An overview of research opportunities. IEEE Internet Things J..

[6] De Brito M.S., Hoque S., Magedanz T., Steinke R., Willner A., Nehls D., Keils O., Schreiner F. A service orchestration architecture for fog-enabled infrastructures. Proceedings of the Second International Conference on fog and Mobile Edge Computing (FMEC).

[7] DDragoni N., Giallorenzo S., Lafuente A.L., Mazzara M., Montesi F., Mustafin R., Safina L. (2017). Microservices: Yesterday, today, and tomorrow. Present and Ulterior Software Engineering.

[8] Perera C., Qin Y., Estrella J.C., Reiff-Marganiec S., Vasilakos A.V. (2017). Fog computing for sustainable smart cities: A survey. ACM Comput. Surv..

[9] ETSI Technical Specification, oneM2M Functional Architecture. http://www.etsi.org/deliver/etsi_ts/118100_118199/118101/02.10.00_60/ts_118101v021000p.pdf.

[10] OpenFog Consortium, OpenFog Reference Architecture for fog computing. https://www.openfogconsortium.org/wp-content/uploads/OpenFog_Reference_Architecture_2_09_17-FINAL.pdf.

[11] ETSI Technical Specification, Mobile Edge Computing (MEC): Framework and Reference Architecture. http://www.etsi.org/deliver/etsi_gs/MEC/001_099/003/01.01.01_60/gs_MEC003v010101p.pdf.

[12] ETSI Technical Specification, Network Functions Virtualisation (NFV): Management and Orchestration. http://www.etsi.org/deliver/etsi_gs/NFV-MAN/001_099/001/01.01.01_60/gs_NFV-MAN001v010101p.pdf.

[13] OMA White Paper, Lightweight M2M: Enabling Device Management and Applications for the Internet of Thing. http://cdn2.hubspot.net/hub/183757/file-610591431-pdf/docs/OMA_Whitepaper_Lightweight_M2M_3-14%5b5%5d.pdf.

[14] Topology and Orchestration Specification for Cloud Applications (TOSCA), OASIS. https://www.oasis-open.org/committees/tc_home.php?wg_abbrev=tosca.

[15] Satyanarayanan M., Bahl P., Caceres R., Davies N. (2009). The case for vm-based cloudlets in mobile computing. IEEE Pervasive Comput..

[16] Sánchez L., Gutiérrez V., Galache J.A., Sotres P., Santana J.R., Casanueva J., Muñoz L. SmartSantander: Experimentation and service provision in the smart city. Proceedings of the 16th International Symposium on Wireless Personal Multimedia Communications (WPMC).

[17] Sanchez L., Muñoz L., Galache J.A., Sotres P., Santana J.R., Gutierrez V., Ramdhany R., Gluhak A., Krco S., Theodoridis E. (2014). SmartSantander: IoT experimentation over a smart city testbed. Comput. Netw..

[18] Puiu D., Barnaghi P., Toenjes R., Kümper D., Ali M.I., Mileo A., Parreira J.X., Fischer M., Kolozali S., Farajidavar N. (2016). Citypulse: Large scale data analytics framework for smart cities. IEEE Access.

[19] Abreu D.P., Velasquez K., Curado M., Monteiro E. (2017). A resilient Internet of Things architecture for smart cities. Ann. Telecommun..

[20] Petrolo R. Towards a smart city based on cloud of things. Proceedings of the 2014 ACM International Workshop on Wireless and Mobile Technologies for Smart Cities.

[21] Liu X., Liu Y., Song H., Liu A. (2017). Big Data Orchestration as a Service Network. IEEE Commun. Mag..

[22] Naeem M., Ejaz W., Karim L., Ahmed S.H., Anpalagan A., Jo M., Song H. (2017). Distributed Gateway Selection for M2M Communication in Cognitive 5G Networks. IEEE Netw..

[23] SONATA NFV: Agile Service Development and Orchestration in 5G Virtualized Networks. http://sonata-nfv.eu/.

[24] COHERENT, a Unified Programmable Control Framework for 5G Heterogeneous Radio Access networks. http://www.ict-coherent.eu/.

[25] SELFNET, Framework for Self-organized Network Management in Virtualized and Software Defined Networks. https://selfnet-5g.eu/.

[26] SLICENET: End-to-End Cognitive Network Slicing and Slice Management Framework in Virtualised Multi-Domain, Multi-Tenant 5G Networks. https://5g-ppp.eu/slicenet/.

[27] SESAME: Small cEllS Coordination for Multi-Tenancy and Edge Services. http://www.sesame-h2020-5g-ppp.eu/.

[28] Openstack, Open Source Software for Creating Private and Public Clouds. https://www.openstack.org/.

[29] OpenBaton, An extensible and customizable NFV MANO-Compliant Framework. http://openbaton.github.io/.

[30] OpenMano, An Open Source Project Providing a Practical Implementation of the Reference Architecture for Management and Orchestration under Standardization at ETSI NFV ISG. https://github.com/nfvlabs/openmano.

[31] OpenDayLight, a Modular Open Platform for Customizing and Automating SDN Networks of Any Size and Any Scale. https://www.opendaylight.org/.

[32] ONOS, An Open Source SDN Network Operating System Designed for Building Next-Generation SDN/NFV Solutions. http://onosproject.org/.

[33] Ryu, Component-Based SDN Framework. https://osrg.github.io/ryu/.

[34] OMA LightweightM2M Specifications and LwM2M Developer Tool Kit. https://github.com/OpenMobileAlliance/OMA_LwM2M_for_Developers.

[35] LESHAN: OMA Lightweight M2M Server and Client Java Implementation. https://github.com/eclipse/leshan.

[36] 5G-EmPOWER: Multi-access Edge Computing Operating System Supporting Lightweight Virtualization and Heterogeneous Radio Access Technologies. http://empower.create-net.org/.

[37] Moy J. OSPF Version 2. RFC Editor, Standards Track, 1997, RFC 2178. https://tools.ietf.org/html/rfc2178.

[38] Latre S., Leroux P., Coenen T., Braem B., Ballon P., Demeester P. City of things: An integrated and multi-technology testbed for IoT smart city experiments. Proceedings of the 2016 IEEE International Smart Cities Conference (ISC2).

[39] Jiang X. (2017). Large Scale Air-Quality Monitoring in Smart and Sustainable Cities. Smart Cities: Foundations, Principles, and Applications.

[40] Pedregosa F., Varoquaux G., Gramfort A., Michel V., Thirion B., Grisel O., Blondel M., Prettenhofer P., Weiss R., Dubourg V. (2011). Scikit-learn: Machine learning in Python. J. Mach. Learn. Res..

[41] Sun W., Choi M., Choi S. (2013). IEEE 802.11 ah: A long range 802.11 WLAN at sub 1 GHz. J. ICT Stand..

[42] Adelantado F., Vilajosana X., Tuset-Peiro P., Martinez B., Melia-Segui J., Watteyne T. (2017). Understanding the limits of LoRaWAN. IEEE Commun. Mag..

[43] Zuniga J.C., Ponsard B. Sigfox System Description. Proceedings of the Internet Engineering Task Force (IETF) Meetings.

